# The Eco-Friendly Biochar and Valuable Bio-Oil from *Caragana korshinskii*: Pyrolysis Preparation, Characterization, and Adsorption Applications

**DOI:** 10.3390/ma13153391

**Published:** 2020-07-31

**Authors:** Tongtong Wang, Hongtao Liu, Cuihua Duan, Rui Xu, Zhiqin Zhang, Diao She, Jiyong Zheng

**Affiliations:** 1 State Key Laboratory of Soil Erosion and Dryland Farming on the Loess Plateau, Northwest A & F University, Yangling 712100, China; tongtwang@163.com (T.W.); liuhongtao@nwafu.edu.cn (H.L.); chduan@nwafu.edu.cn (C.D.); 2019055450@nwafu.edu.cn (R.X.); zhangzhiqin@nwafu.edu.cn (Z.Z.); diaoshe888@163.com (D.S.); 2 College of Natural Resources and Environment, Northwest A & F University, Yangling 712100, China; 3 Institute of Soil and Water Conservation, Chinese Academy of Sciences and Ministry of Water Resources, Yangling 712100, China

**Keywords:** pyrolysis process, *Caragana korshinskii* biochar, physicochemical properties, adsorption characteristics, nitrate nitrogen, bio-oil

## Abstract

Carbonization of biomass can prepare carbon materials with excellent properties. In order to explore the comprehensive utilization and recycling of *Caragana korshinskii* biomass, 15 kinds of *Caragana korshinskii* biochar (CB) were prepared by controlling the oxygen-limited pyrolysis process. Moreover, we pay attention to the dynamic changes of microstructure of CB and the by-products. The physicochemical properties of CB were characterized by Scanning Electron Microscope (SEM), BET-specific surface area (BET-SSA), X-ray photoelectron spectroscopy (XPS), X-ray diffraction (XRD), Fourier Transform Infrared (FTIR), and Gas chromatography-mass spectrometry (GC-MS). The optimal preparation technology was evaluated by batch adsorption application experiment of NO_3_^−^, and the pyrolysis mechanism was explored. The results showed that the pyrolysis temperature is the most important factor in the properties of CB. With the increase of temperature, the content of C, pH, mesoporous structure, BET-SSA of CB increased, the cation exchange capacity (CEC) decreased and then increased, but the yield and the content of O and N decreased. The CEC, pH, and BET-SSA of CB under each pyrolysis process were 16.64–81.4 cmol·kg^−1^, 6.65–8.99, and 13.52–133.49 m^2^·g^−1^, respectively. CB contains abundant functional groups and mesoporous structure. As the pyrolysis temperature and time increases, the bond valence structure of C 1s, Ca 2p, and O 1s is more stable, and the phase structure of CaCO_3_ is more obvious, where the aromaticity increases, and the polarity decreases. The CB prepared at 650 °C for 3 h presented the best adsorption performance, and the maximum theoretical adsorption capacity for NO_3_^−^ reached 120.65 mg·g^−1^. The Langmuir model and pseudo-second-order model can well describe the isothermal and kinetics adsorption process of NO_3_^−^, respectively. Compared with other cellulose and lignin-based biomass materials, CB showed efficient adsorption performance of NO_3_^−^ without complicated modification condition. The by-products contain bio-soil and tail gas, which are potential source of liquid fuel and chemical raw materials. Especially, the bio-oil of CB contains α-d-glucopyranose, which can be used in medical tests and medicines.

## 1. Introduction

The *Caragana korshinskii* Kom. (1909) is a legume shrub that is widely distributed across desert habitats with gravely, sandy, and saline soils in arid and semi-arid areas of Asia and Africa [1]. Many scholars believe that it may be an ideal biomass energy crop with high heating value, strong drought tolerance, and high sprouting capacity [2]. As a pioneer species of arid areas in northern China, the *Caragana korshinskii* is essential for windbreak function, sand fixation, and water and soil conservation [3]. The planting area of *Caragana korshinskii* is conservatively estimated more than 1.6 million km² [4]. However, because of the lack of corresponding development technologies and products worldwide, the utilization rate of *Caragana korshinskii* resources is less than 40% and even a nearly null rate in some areas. Other countries or regions also produce a large number of *Caragana korshinskii* yearly, which has been regarded as a new forestry wastes [5]. They represent not only a waste of energy but also a threat to the local environment [6,7]. Therefore, developing the new technology and product and enhancing the efficient use of *Caragana korshinskii* resources are necessary and urgent. It cannot only help local farmers solve waste problems and promote environmental sustainability [8], but also produce positive economic benefits in arid areas of Asia and Africa which are usually relatively underdeveloped areas.

The high temperature pyrolysis is generally the most common and important carbonization method [9]. Under limited oxygen supply or anoxic conditions and at a certain temperature (<700 °C), biomass resources can be carbonized into biochar, a carbon-rich, stable, and highly aromatic solid product [10,11,12,13]. At present, because of its large specific surface area, high stability, strong adsorption capacity, porous structure, as well as its wide range of raw materials, and low cost, biochar was widely used in agricultural and environmental protection fields such as pollution remediation, modified materials, soil improvement, etc., [14,15]. The carbonization process is mainly controlled by parameters such as pyrolysis temperature, time, and heating rate. It was a general consensus that pyrolysis process had great influence on the physical and chemical properties of biochar. Chen et al. [16] and Fan et al. [17] set different pyrolysis temperatures to study the properties of biochar, and found that the pyrolysis temperature had a vital impact on the carbonization process of raw materials, and direct influence on the yield, element content, surface functional group, and pore size distribution. Higher pyrolysis temperatures had a positive effect on the adsorption applications. Xue et al. [18] observed that the biochar yield rate ranged from 89.7% to 51.2% at temperature from 300 to 600 °C. The surface characteristics and micromorphology of biochar changed with increasing pyrolysis temperature and time, but the heating rate has little effect on the properties of biochar [19]. Biomass pyrolysis usually also produces by-products, bio-oil, and tail gas. High temperature heating of the biomass in the pyrolysis process caused irregular thermal movement in the internal molecular structure of the biomass and eventually decomposition into small molecule polymer [20]. Both biochar and by-products can be generally well-obtained at conventional heating rate of 10 °C·min^−1^ [21].

However, some reports on biomass pyrolysis are not systematic. Most scholars pay more attention to yield data and basic properties than the changes of bio-oil and tail gas during pyrolysis process. The research on the effects of microscopic indexes such as XPS, XRD, and FTIR is also not sufficient and deep enough [22]. In order to better promote the application of biomass pyrolysis technology, it is meaningful to systematically study the impact of pyrolysis process on its properties [23,24]. Moreover, nitrate nitrogen (NO_3_^−^) is the main form of nitrogen, which plays an important role in plant nutrition, microbial transformation, and natural nitrogen cycle [25]. But the nitrogen leaching from farmland will lead to many problems such as groundwater pollution and water eutrophication [26]. Fortunately, the adsorption of NO_3_^−^ by biochar is the most effective method to remove nitrogen because of high efficiency, simple equipment, and reliable operation [27]. Indeed, NO_3_^−^ was selected as the object for adsorption application, which has always been a research hotspot [28]. Li et al. [25] found that biochar has limited adsorption of NO_3_^−^ from switchgrass and water oak at variable pyrolysis condition. Even if the biochar is modified by FeCl_3_ impregnated, the maximum adsorption capacity for nitrogen is only 14 mg·g^−1^ [29]. Therefore, *Caragana korshinskii* was used as the raw material to prepare 15 kinds of biochar by oxygen-limited pyrolysis with controlled pyrolysis temperature and time. Some characterization methods were used to investigate the effects of different pyrolysis processes on the changes of biochar properties. At the same time, the by-products are systematically analyzed for chemical composition, and the pyrolysis mechanism is explored. The adsorption characteristics of NO_3_^−^ in aqueous solution were studied by batch adsorption experiment. This work will provide a theoretical and practical basis for the efficient utilization of *Caragana korshinskii* resource and development technology and products about the eco-friendly biochar and valuable by-products.

## 2. Materials and Methods 

### 2.1. Production of Biochar and By-Products

**Figure 1:**
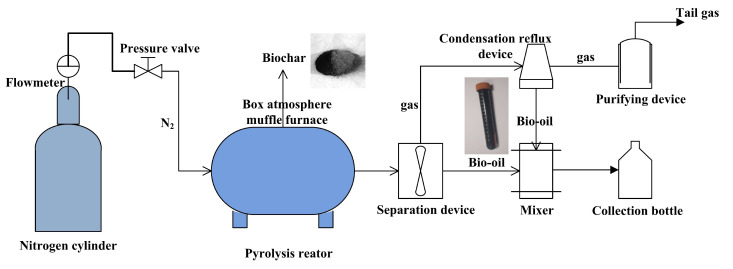
Schematic for pyrolysis process of *Caragana korshinskii* biomass.

The *Caragana korshinskii* samples (20 years old) were collected from the Shanghuang Village, Guyuan City, Ningxia Hui Autonomous Region, China. The ratios of cellulose, hemicellulose, and lignin in the biomass of *Caragana korshinskii* are approximately 45%, 20%, and 35% respectively. The biomass was cut lengthwise into 10-cm pieces, washed with distilled water several times, and dried. Subsequently, a box was filled with the samples, sealed with a cover, and placed into a GF11Q-B box atmosphere muffle furnace (Nanjing Boyuntong Instrument Technology Co., Ltd., Nanjing, China). The pyrolysis temperatures were set to 450, 500, 550, 600, and 650 °C. The pyrolysis time (maintain the constant temperature) was set to 2, 3, and 4 h, and the constant heating rate to 10 °C·min^−1^. Nitrogen gas was used to protect the anaerobic environment. A three-stage temperature-programmed pyrolysis method was used to uniformly cool the samples to room temperature. The bio-oil and tail gas were collected by a condensing reflux device (Figure 1). Subsequently, the *Caragana korshinskii* biochar (CB) was crushed into small pieces of approximately 2 cm by a pulveriser (Zhejiang Fengli Pulverization Equipment Co., Ltd., Shaoxing, China).

### 2.2. Characterisation and Test

#### 2.2.1. Basic Properties

The element contents of C, H, O, and N were measured using a Vario EL cube elemental analyzer (Element Analysensysteme Co., Ltd., Berlin, Germany) with argon as the carrier gas. The pH of the CB samples were determined by mixing 1.00 g of biochar and 20.0 mL of deionized water for 1 h in a rotary shaker. After that, the mixture was still at room temperature for 1 h, and its pH was measured by a PHS-3C pH meter (Rex INESA Scientific Instrument Co., Ltd., Shanghai, China). According to Li et al. [21], the barium chloride-sulfuric acid-forced exchange method was used to determine the CEC of CB, and the yield of CB is equal to the ratio of the mass of biochar produced by pyrolysis to the mass of original dried *Caragana korshinskii*.

#### 2.2.2. Microscopic Indexes

CB samples of approximately 0.5 g were degassed for 3 h at 125 °C. The surface morphology of the CB samples was analyzed in a JSM-6510LV scanning electron microscope (JEOL Ltd., Tokyo, Japan) at a 20 kV. The BET-SSA and pore size analysis were determined using N_2_ as the adsorbate at 77 K and relative pressure of 0.05–0.20, for which a V-Sorb 2800P BET-SSA and pore size analyzer (Gold APP Instrument Co., Ltd., Beijing, China) was used. All CB samples were scanned with an Escalab 250 Xi X-ray photoelectron spectrometer (Thermo Fisher Scientific Co., Ltd., Waltham, MA, USA) to investigate the chemical state of the main elements and the functional groups on the surface. The chemical bonds were identified by published references and the National Institute of Standards and Technology database (https://srdata.nist.gov/xps/). The mineral species of the CB samples were identified using a D/max2400 X-ray powder diffractometer (Rigaku Co., Ltd., Wilmington, NC, USA) at a 0.02 scan step size, 2 deg·min^−1^ scan speed, 0.15 receiving slit width, 30–40 kV, and 30–40 mA. The surface functional groups of CB samples were measured with KBr pellet methods by infrared spectra analysis using a Vertex70 FTIR spectrometer (Bruker Co., Ltd., Billerica, USA) for 16 scans over a range of 400–4000 cm^−1^ with a resolution of 2 cm^−1^.

#### 2.2.3. By-Product Analysis

The gas chromatography tandem mass spectrometer (GC-MS, Agilent 7890A/7000B GC-QQQ, Santa Clara, CA, USA) was used to analyze the components of bio-oil and tail gas. GC-MS analysis conditions are: Shunt injection with a ratio of 25:1, HP-5 (30 m × 0.25 mm × 0.25 m) column, the temperature of vaporization chamber at 260 °C, high purity helium as carrier gas, and flow rate 1.0 mL/min. The column temperature is programmed: the initial temperature is 50 °C and kept for 5 min, the temperature is raised to 100 °C at 2 °C/min, and then 180 °C at 1 °C/min, finally 260 °C at 1 °C/min and kept for 5 min. MS electron energy was 70 eV, filament current 80 A, ion source temperature 230 °C.

### 2.3. Batch Experiments

The biochar sorption capacity was evaluated by batch experiments. KNO_3_ was used to prepare standard NO_3_^−^ solutions at different concentrations. Accurately weighted samples of 0.1000 g, including above 15 kinds of CB, were placed in 250 mL conical flasks and mixed with 50 ml of the NO_3_^−^ solution. The concentration of NO_3_^−^ in the solution is 50 mg·L^−1^. Moreover, the background electrolyte was 0.01 mol·L^−1^ KCl. The conical flasks were sealed with a plastic film and placed into an air bath constant temperature oscillator at 25 ± 1 °C, which was shaken at 150 rpm for 3 h. The obtained suspension was filtered with a 0.45 μm microfiltration membrane. The concentrations of NO_3_^−^ in the filtrate was considered the equilibrium concentrations of the solutions, and measured using a continuous flow auto analyzer (Bran+luebbe, Analyzer 3-AA3, Hamburg, Germany). All experiments were performed in triplicate with appropriate blanks (biochar and H_2_O), and statistical methods were used to analyse the mean values. The adsorption capacity (*Q_e_*, mg·g^−1^) and the removal efficiency (*RE*, %) were calculated according to the following equations:

(1) $$Q_{e}=\left(C_{0}-C_{e}\right)\frac{V}{m}$$

(2) $$RE=1-\frac{C_{e}}{C_{0}}\times 100\%$$

Throughout this paper, *Q_e_* is the adsorption capacity at equilibrium, and *C_e_* and *C*_0_ are the equilibrium and initial NO_3_^−^ concentrations (mg·L^−1^), respectively. *V* is the volume of the solution (L), and *m* is the mass of added biochar (g). The following steps were used for batch experiments using optimal CB (alternative biochar). (1) Isothermal adsorption: the initial concentration gradients of NO_3_^−^ were set to 0, 2, 5, 10, 20, 30, 40, 50, 60, 70, 80, 90 and 100 mg·L^−1^; (2) adsorption kinetics: the adsorption times of NO_3_^−^ were set to 5, 10, 15, 30, 60, 180, 360, 540, 720, 1080, and 1440 min, other settings are the same.

### 2.4. Adsorption Models and Data Analysis

To better investigate the characteristics of the adsorption, four isotherms models (Langmuir, Freundlich, Temkin, and Dubinin-Radushkevich) [11] and four kinetic models (pseudo-first-order, pseudo-second-order, Elovich, and intraparticle diffusion model) [30] were used, as shown in Table 1. In this work, data statistics were calculated using Excel 2016 Pro (Microsoft, Redmond, WA, USA) and SPSS 20.0 (IBM Corporation, Armonk, NY, USA). One-way Analysis of Variance (ANOVA) using SPSS 20.0 was conducted to compare the means of the measured values at *p* < 0.05 for each treatment. Isothermal adsorption and adsorption kinetic curves were fitted and plotted using OriginPro 8.5.1 SR2 (OriginLab, Northampton, MA, USA). In addition, XRD was performed using Jade 6.5 (Materials data Inc., Livermore, CA, USA) to determine the mineral composition of biochar. Wide XPS scans were analyzed using Avantage v5.979 (Thermo Fisher Scientific Co., Ltd., Waltham, MA, USA) to determine the surface elements content. Narrow scans of C1s were fitted using Avantage v5.979 to analyze and quantify the existing state of C.

Where *Q_e_* and *Q_t_* are the adsorbed capacity (mg·g^−1^) at an equilibrium concentration (*C_e_*, mg·g^−1^) and a given time of NO_3_^−^ in solution, respectively. *Q_m_* (mg·g^−1^) denotes the maximum adsorption capacity. *a* and *K_F_* are the Langmuir (mg·L^−1^) and Freundlich (mg·g^−1^) constants, respectively. 1/*n* is the heterogeneity factor. *A* is the equilibrium binding constant (mg·L^−1^), and *B* is the Temkin constant related to the adsorption heat. *β* is the D-R model coefficient (mol^2^·J^−2^), *Q*_0_ is the maximum unit adsorption capacity (mmol·g^−1^), *ε* is the Polanyi adsorption potential, *R* is the ideal gas constant 8.314 J·(mol·K)^−1^, *T* is the absolute temperature, and *E* is the adsorption free energy (J·mol^−1^). *k*_1_, *k*_2_, and *k_i_* are the rate constants for the pseudo-first-order (min^−1^), pseudo-second-order (g·mg^−1^·min^−1^), and the intraparticle diffusion (mg·g^−1^·h^−1/2^) rate constant, respectively. *h* is the initial adsorption rate (mg·g^−1^·min^−1^). *a*′ is the initial sorption ratio (g·mg^−1^·min^−1^), and *b* is the desorption constant (g·mg^−1^), which is related to the extent of surface coverage and activation energy for chemisorption. *C* is a constant indicating the number of boundary layers of the adsorbent.

## 3. Results and Discussion

### 3.1. Basic Physicochemical Properties

The physicochemical properties of CB in all processes are shown in Table 2. With the increase of pyrolysis temperature, the content of C in biochar increases continuously, while the content of O and N decreases gradually, which is consistent with many research results [31,32]. This is because they are precipitated in the form of small molecule organic matter and water during the pyrolysis process [18,33]. As an important index of elemental analysis, the O/C has been used to determine the aromatic structure and composition of polymer, and the N/C can reflect the metabolic decomposition of microorganisms, and the (O + N)/C can be used to characterize the polarity of adsorbent. It can be clearly seen from Table 2 that O/C, N/C, and (O + N)/C decrease obviously with the increase of temperature, which is mainly due to the intensification of dehydrogenation and deoxidation reactions during the pyrolysis process. But, at the same temperature, O/C, N/C, and (O + N)/C change irregularly with the pyrolysis time. The change in O/C shows that the increase of temperature can promote the fracture of weak chemical bonds and the formation of condensation products, so the CB prepared at high temperature is more stable. In addition, O/C can also characterize the CEC of CB, and the larger the O/C, the larger the CEC [34]. In this work, the N/C of 15 kinds of CB is not high, which indicates that it is easy to decompose and will not consume the available nitrogen when applied to the soil [25]. The results in (O + N)/C shows that the oxygen-containing functional groups such as hydroxyl, carboxyl, and carbonyl groups of CB may be burned out with the increase of temperature, that is, the polar functional groups decreases and the hydrophobicity of biochar increases.

The pH of CB increases with the increase of pyrolysis temperature. For pyrolysis time, the average pH is 8.38 for 2 h, 8.51 for 3 h, and 8.58 for 4 h. With the increase of time, the pH of the obtained CB increases. The pH of the CB under each pyrolysis conditions was 6.65–8.99, which was close to other lignin-based biochar [35]. Singh et al. [36] found that the biochar pH ranged from 6.93 to 10.26. This is due to the gradual separation of alkali salts from the biomass structure and the increase of alkaline functional groups on the surface of biochar [37], which is mutually supportive of the findings by Cantrell et al. [38]. Cantrell et al. [38] believed that the inorganic minerals (alkaline salts) in biochar were the main reason for the alkaline pH of biochar, and the oxygen-containing functional groups on the surface of biochar may also have certain contribution to the pH. Shaaban et al. [34] also believed that the biochar at high temperature had less acidic volatiles and more ash than low temperature, so the pH of biochar was higher. In addition, prolonging the pyrolysis time will help the further development of biochar.

The average statistics of 2 h and 3 h showed that the CEC of CB decreased first and then increased as the increase of temperature from 450 to 650 °C. The CEC of CB for 4 h decreased with the increase of temperature. For the pyrolysis time, the average CEC was 45.34 cmol·kg^−1^ for 2 h, 44.31 cmol·kg^−1^ for 3 h, and 41.47 cmol·kg^−1^ for 4 h. The CEC of CB under each pyrolysis conditions is 16.64–81.4 cmol·kg^−1^. In the pyrolysis process, the loss rate of some acidic functional groups of biomass is the fastest at low temperature but slows down after 450 °C. The general trend is to decrease with the increase of temperature. Guo et al. [39] also believed this phenomenon of the CEC decrease. But, Liang et al. [40] found that CEC increased with the increase of temperature by studying the pyrolysis process of pine wood. In this work, the CEC only increased at 650 °C.

On average, the yield of CB was 32.22% at 450 °C, followed by 31.53% at 500 °C, 29.67% at 550 °C, 27.23% at 600 °C, and 25.90% at 650 °C. This shows that even if the temperature rises slightly at 50 °C, the yield will obviously decrease by 2–8%, especially in the low temperature region. It may be because pyrolysis at lower temperatures promotes the degradation of organic components of raw materials and produces various volatile substances and high-boiling substances, while at high temperatures, high-boiling and non-volatile substances decompose slowly [34,41]. Pyrolysis time also has a significant effect on the yield of CB. The average yield of CB was 29.65% at 2 h, and then decreased, 29.55% at 3 h, and 28.74% at 4h. It can be obviously concluded that with the increase of pyrolysis time, the yield of CB decreases, and the decrease range of yield is about 1.5% for every 1 h of increase. Compared with the pyrolysis temperature, the effect of pyrolysis time is not significant, which is mainly because the heating rate for preparing CB is constant in this work, that is, the time stays for a long time, but the temperature cannot reach, and the decomposition of high-boiling and non-volatile substances is slow. This is consistent with the results reported by Xue et al. [18].

### 3.2. Biochar Characterization

#### 3.2.1. SEM

**Figure 2:**
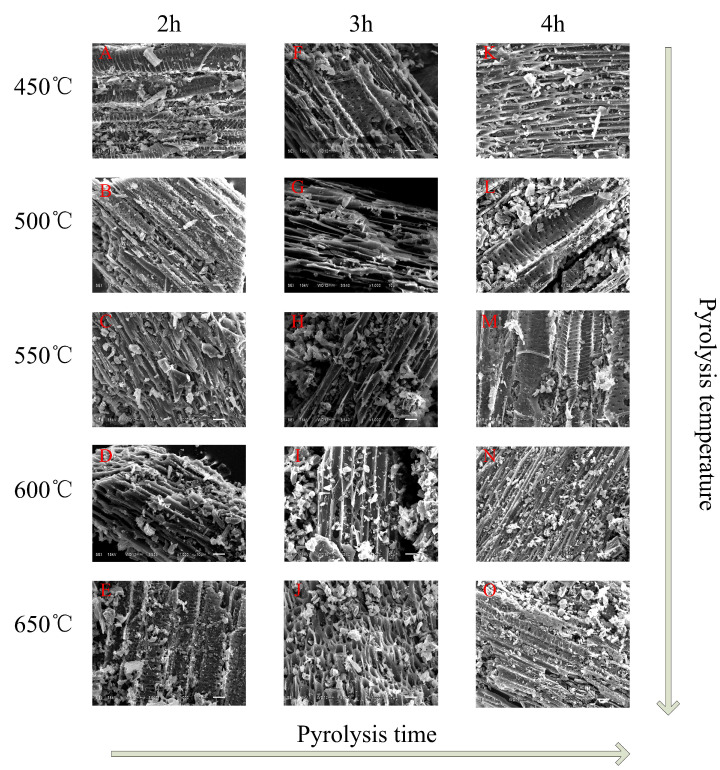
The scanning electron microscope (SEM) images of CB at 1000 times. (**A**) 450 °C/2 h, (**B**) 500 °C/2 h, (**C**) 550 °C/2 h, (**D**) 600 °C/2 h, (**E**) 650 °C/2 h, (**F**) 450 °C/3 h, (**G**) 500 °C/3 h, (**H**) 550 °C/3 h, (**I**) 600 °C/3 h, (**J**) 650 °C/3 h, (**K**) 450 °C/4 h, (**L**) 500 °C/4 h, (**M**) 550 °C/4 h, (**N**) 600 °C/4 h, (**O**) 650 °C/4 h.

The SEM images of the different processes biochar are shown in Figure 2 and Figure S1. It can be seen that CB has obvious pore structure under different carbonization conditions, but the number and size of pores are different. From Figure 2A–E, the microscopic morphology of CB shows flaky irregular polygons with uneven distribution of some broken particles, and there are more loose pores and thin sheets of tubular structure. With the increase of pyrolysis temperature, the surface roughness further increases, and a large number of micropores begin to appear, similar to honeycomb-shaped porous bodies. These honeycomb-shaped micropore structures vary in size and shape, and are closely arranged. From 450 to 650 °C, the more obvious the surface pores texture is, the rougher the surface roughness is, the better the micropore structure is, the thinner the pore wall becomes, and the more the number of pores is. There are the similar rules in Figure 2F–J and Figure 2K–O. These pores are an important basis for biochar to have larger surface area and stronger adsorption performance [18,42]. Comparing Figure 2A,F,K, it is found that the images are very similar, which shows that pyrolysis time has little effect on the pore structure of CB. Further EDS elementary analysis (Figure S1) confirmed that CB not only has higher amounts of elemental C and O, but also Si (0.14%), Cl (3.49%), K (0.3%), Ca (1.19%), and other elements.

#### 3.2.2. BET Specific Surface Area and Pore Size Analysis

**Figure 3:**
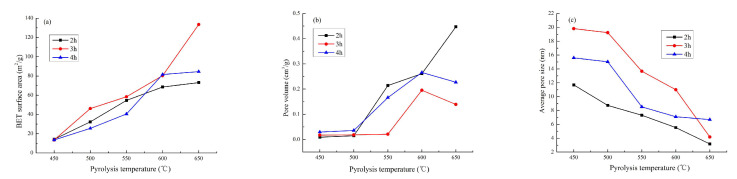
Effects of different pyrolysis processes on BET surface area and pore size analysis of *Caragana korshinskii* biochar (CB). (**a**) BET surface area, (**b**) pore volume, (**c**) average pore size.

The effects of different pyrolysis processes on BET-SSA, pore volume, and pore size of CB are detailed in Figure 3. As shown in Figure 3a, the BET-SSA of CB prepared ranges from 13.52–133.49 m^2^·g^−1^. With the increase of pyrolysis temperature, the BET-SSA of CB increases. It showed that even if the temperature rises by 50 °C, the BET-SSA will increase significantly in the range of 21–61%, especially in the low temperature regions. At the same temperature, the effect of pyrolysis time on the BET-SSA increases first, then decreases, or remains unchanged. The BET-SSA at 650 °C/3 h (Table S1) reached the maximum (133.49 m^2^·g^−1^), which was an ideal alternative biochar. Because of the increase of pyrolysis temperature, the carbon content and the adsorption of hydrophobic pollutants increases while the others decrease, including the oxygen content, the O/C, the hydrophilicity, and polarity of biochar and the affinity for water molecules, resulting in larger specific surface area along with stronger adsorption effect [18,43]. It can be clearly seen from Figure 3b,c that the pore volume increases with the increase of pyrolysis temperature, which has the same trend as the BET-SSA. The average pore size decreases with the increase of pyrolysis temperature. It implies that the large pores on the surface can become mesopores and then develop into microporous structures. At the same temperature, pyrolysis time has little effect on the pore volume and pore size of CB. It is because as the temperature increases, the micropore structure develops, the average pore diameter decreases, the pore wall becomes thinner, the pore number and volume increase [44], and the specific surface area increases.

According to the definition of the International Union of Pure and Applied Chemistry [45], when a pore size is between 2 and 50 nm, it is called mesopores. It means that the pore structure of CB is mainly mesopores (Figure 3c and Figure S2). Pyrolysis temperature has a great influence on the BET-SSA and pore structure of CB, followed by pyrolysis time, which is basically consistent with the results of SEM image observation. Many reports have found that the BET-SSA of biochar decreases at 700 °C [17,25], which is attributed to the evolution of the volatile phenol bubbles, the structural sequence, the decrease in the number of micropores, and the increase in the number of macropores. In view of this change trend, the pyrolysis temperature of 700 °C was not set in this work.

#### 3.2.3. XPS and XRD

**Figure 4:**
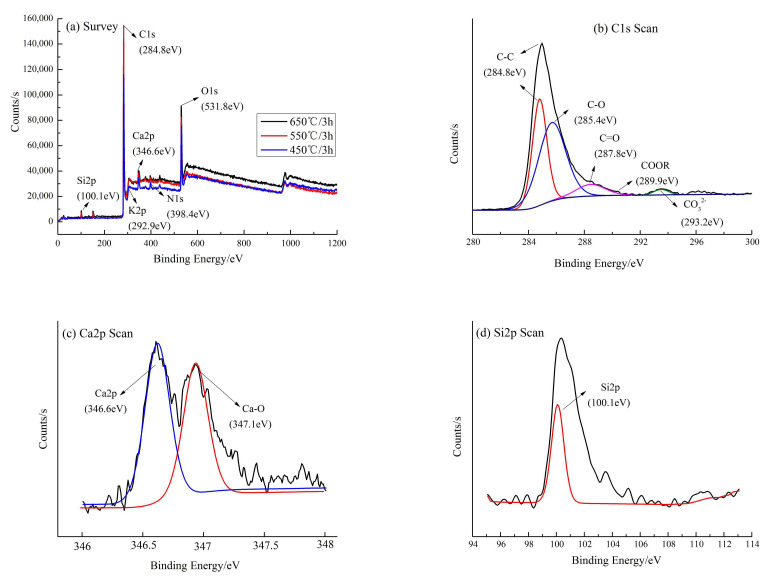
X-ray photoelectron spectra of CB. (**a**) A typical survey scan under different pyrolysis processes (taking 3 h as an example); (**b**) C1s binding energy at 650 °C/3 h; (**c**) Ca2p binding energy at 650 °C/3 h; (**d**) Si2p binding energy at 650 °C/3 h.

The surface of CB was investigated using XPS (Figure 4), and the results indicated six main elements, namely C, O, N, Si, K, and Ca, and two minor elements, namely Fe and Cl. Their average mass percentages were 74.55%, 18.73%, 0.41%, 3.92%, 0.72%, 0.45%, 0.42%, and 0.30%, respectively. The photoelectron lines with binding energy (*E_B_*) of approximately 100.1, 284.8, 292.9, 346.6, 398.4, and 531.8 eV were attributed to Si 2p, C 1s, K 2p, Ca 2p, N 1s, and O 1s, respectively (Figure 4a). Detailed XPS data for the C 1s, Ca 2p and Si 2p core level spectrum with a peak fitting of its envelope are presented in Figure 4b–d. The C1s XPS spectrum can be curve fitted into four peak components at approximately 284.4 eV (C–C), 285.4 eV (C–O), 287.8 eV (C=O), and 289.9 eV (C-OOR). In addition, according to the relevant literature, there may be a peak at approximately 293.2 eV (CO_3_^2−^) attributed to the high cellulose, hemicelluloses, and lignin contents of *Caragana korshinskii*. With the increase of temperature, the bond valence structure of each element slowly appears and tends to be stable, and the peak value becomes higher with the increase of temperature.

**Figure 5:**
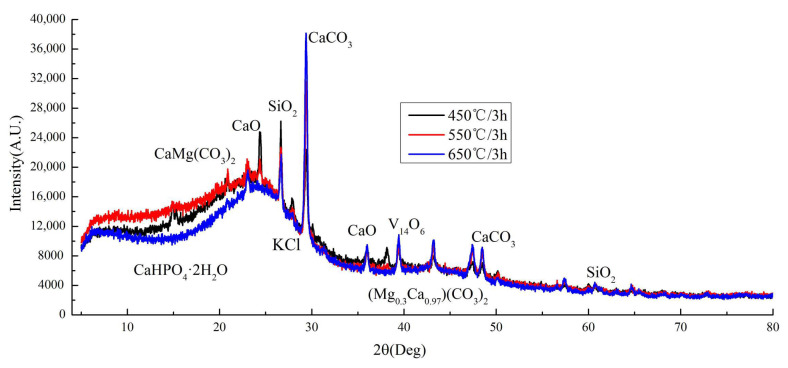
XRD patterns of CB biochar under different pyrolysis processes (taking 3 h as an example).

The XRD analysis (Figure 5) revealed that the principal diffraction peaks at low 2θ angles were sharp and symmetric, indicating the presence of mineral crystals. According to the Jade 6.5 PDF cards [46], the two strong peaks at 26.6 and 29.5° suggest the presence of SiO_2_ and CaCO_3_, respectively. Other strong peaks which suggested CaO, KCl, and CaHPO_4_·2H_2_O were observed on the CB’s surface. Moreover, there was a small peak of (Mg_0.3_Ca_0.97_)(CO_3_) with a highly crystalline structure. As the pyrolysis temperature increases, the crystallization of CaO, KCl, CaHPO_4_·2H_2_O and MgCa(CO_3_) gradually transforms, and the peaks of the XRD pattern decrease, or tend to be flat. The peaks of SiO_2_ and CaCO_3_ become more obvious, indicating that pyrolysis temperature increase may be beneficial to the particle growth of inorganic deposits and thus promote the formation of crystallization [34,38]. It means that the cellulose and lignin components in CB gradually degrade, and release a lot of ash. The ash was mainly composed of mineral components such as silicate, potassium salt, and calcium carbonate. Fan et al [17] believed the calcium oxalate decomposed when the temperature raised above 500 °C, the ash content decreased, and calcium carbonate crystals gradually formed. Thus, the XRD results are in good agreement with the XPS analysis (Figure 4c,d). At the same temperature, pyrolysis time has little effect on the XPS and XRD of CB (Figure 4a and Figure 5). 

#### 3.2.4. FTIR

**Figure 6:**
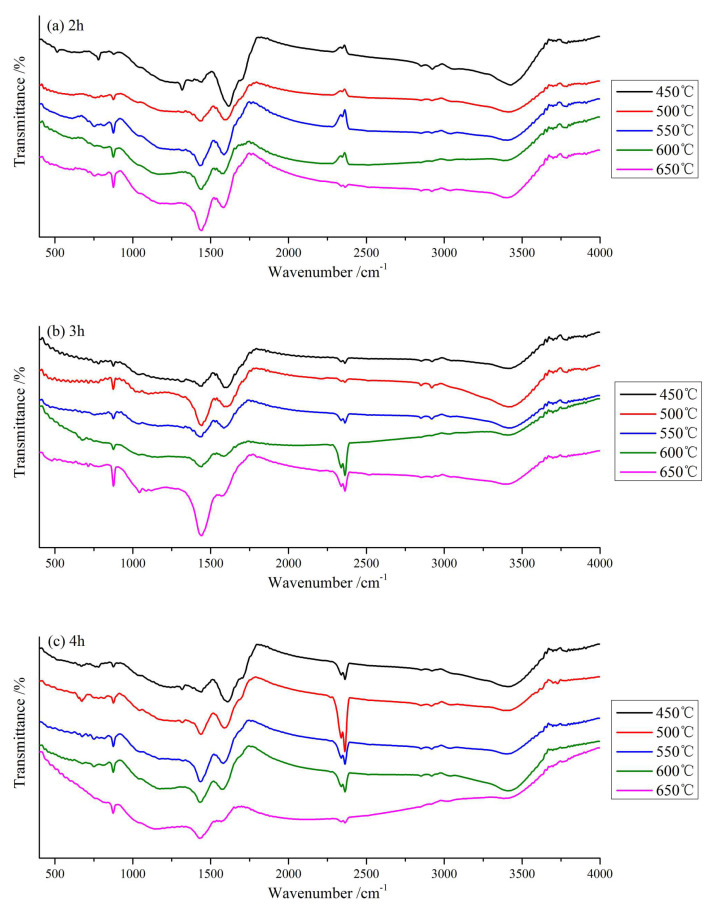
FTIR spectrograms of CB at different pyrolysis processes. (**a**) 2 h; (**b**) 3 h; (**c**) 4 h.

The FTIR of CB is shown in Figure 6. It can be clearly seen that CB has about six identical peak positions, which are located near 500–900, 1300–1400, 1600, 2300, 2800, and 3400 cm^−1^. Viewing the spectrum data table [3,6,11,17,18,24], it was found that CB has -OH vibration absorption peak (3418 cm^−1^), and the C-H in alkanes are mainly vibration absorption peaks of methyl and methylene groups (2854–2966 cm^−1^), the fatty C-H and C=O groups (2280 cm^−1^), aromatic acids -COOH groups (1697 cm^−1^), amide stretching vibration -C=O group (1650 cm^−1^), NH_4_^+^ group (1396 cm^−1^), the pyridine and indole of aromatic compounds (500–900 cm^−1^) groups. It shows that CB has rich functional groups on its surface. Qualitatively, the peak positions of the 15 kinds of CB are roughly the same, and the peak curves are similar, indicating that the functional group types are basically the same. There are certain differences in the surface functional groups of biochar prepared at different temperatures. With the temperature increases, the total content of surface functional groups of biochar decreases, which is consistent with the related research results [19].

Comparing Figure 6a–c, we found that with the increase of temperature, the absorption peak near 3420 cm^−1^ gradually weakens, indicating that the -OH group decreases, which is probably due to the gradual breakage of -OH group by hydrogen-bonded and the separation of bound water [18]. At 2941 cm^−1^, the absorption peak of C-H vibration in alkanes decreases, which means the loss of alkyl group of CB and the aromatization degree of CB gradually increases with the increase of temperature. Among them, the methylene group around 2927 cm^−1^ is gradually degraded or changed. For the fatty C-H and C=O groups of 2280 cm^−1^, the absorption peak gradually increases, especially in Figure 6b, which reached the maximum at 650 °C/3 h. With the increase of pyrolysis temperature, the biochar components have experienced the transition of excessive char, amorphous char, composite char, and chaotic char in turn [27]. The aromatic acid -COOH group (1697 cm^−1^) and amide stretching vibration -CON- group (1650 cm^−1^) gradually decrease, and even disappear in some scientific reports [32,41]. In the characteristic zone of benzene rings around 1400 cm^−1^, the absorption peak gradually increased, indicating that the aromatization degree was increased. On the whole, the functional groups of biochar with different pyrolysis processes have certain differences, mainly showing that with the increase of pyrolysis temperature, the content of basic functional groups (benzene ring and pyridine) increases, while the content of acidic functional groups (phenolic hydroxyl and carboxyl) decreases, this meaning the aromaticity increases and the polarity decreases. At the same time, new functional groups appeared around 500–900 cm^−1^, which means that an aromatic ring is formed, and the aromatization degree is further enhanced. In addition, we found that the pyrolysis time has basically no effect on the biochar surface functional groups, which is basically consistent with many research results [47,48].

### 3.3. Adsorption Application

#### 3.3.1. Screening for Optimal Preparation Conditions

**Figure 7:**
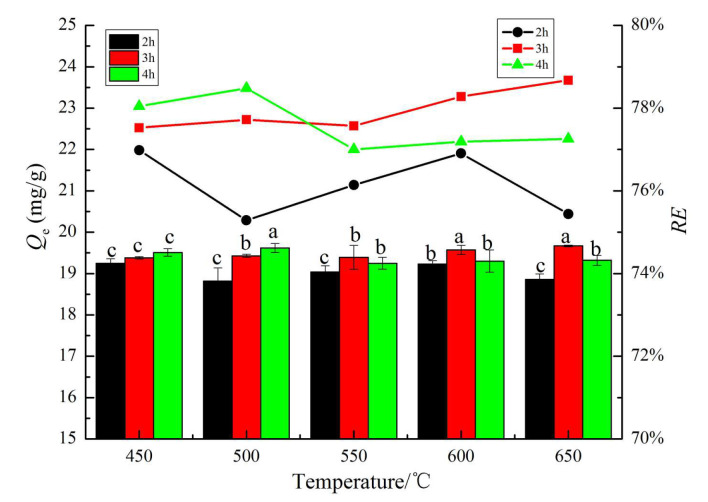
Effects of different pyrolysis process on CB adsorption capacity and removal rate for NO_3_^−^.

The effects of CB prepared under different pyrolysis processes on the adsorption capacity and removal rate of NO_3_^−^ are shown in Figure 7. It can be seen that the adsorption capacity of NO_3_^−^ by CB prepared at 650 °C and 3 h was the largest, reaching 19.67 mg·g^−1^, and the corresponding *RE* reached 78.67% (NO_3_^−^ concentration 50 mg·L^−1^). It is mutually verified by BET-SSA data of CB at 650 °C for 3 h. With the increase of pyrolysis temperature and time, the adsorption capacity and *RE* of NO_3_^−^ by CB showed a rising trend, but the curve presented twists and turns. On average, regardless of the preparation process, the adsorption capacity of CB for NO_3_^−^ is above 18.8 mg·g^−1^, and the removal rate is above 76%, which implies that the *Caragana korshinskii* is an excellent and low-cost biomass raw material. Therefore, CB prepared at 650 °C for 3 h was the optimal biochar and the most appropriate preparation conditions.

#### 3.3.2. Isotherms Adsorption

**Figure 8:**
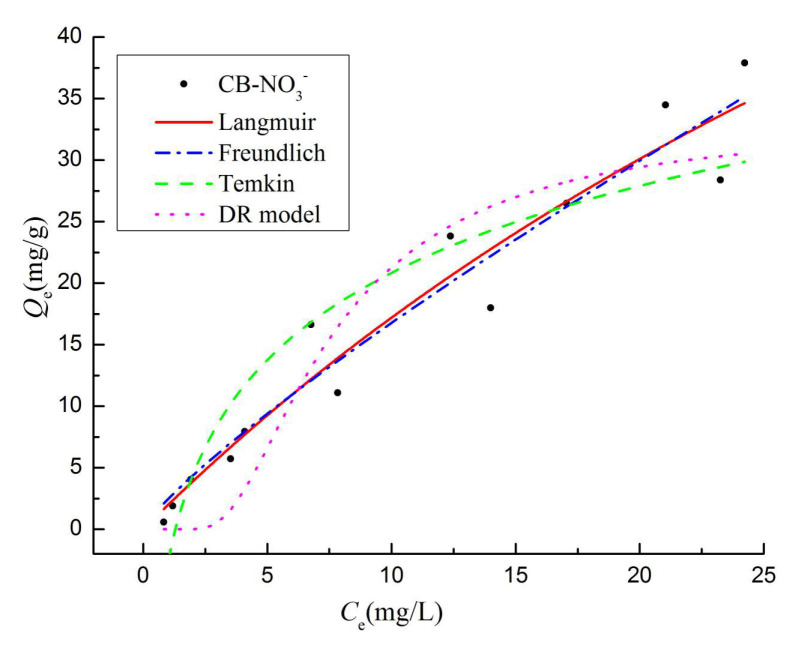
The isotherm adsorption of CB biochar for NO_3_^−^ (650 °C/3 h).

As shown in Figure 8, with increasing initial NO_3_^−^ concentration, the adsorption capacity of CB gradually increased until saturation. At low initial concentrations, the adsorption curve is steep, and the adsorption capacity increases rapidly in a straight line. At high initial concentrations, the adsorption increased slowly and eventually stabilized to reach equilibrium. The initial concentration of the solution is essential to overcome the mass transfer resistance between the liquid and the solid phase. So, higher initial concentration of the solution improves the adsorption capacity of the biochar [49]. The related parameters calculated from the four isotherms models are listed in Table 3. The isotherm models reproduced the adsorption data well. However, the Langmuir models for NO_3_^−^ (highest *R*^2^ value of 0.9364) matched the experimental data better than the other models. These results suggest that the adsorption process is similar to a monolayer adsorption for NO_3_^−^ and mainly physical adsorption [4]. But the Freundlich models presented high fitting decision coefficients, which may also imply that the process involved multilayer complex and associated with chemical interactions [11,15]. In the Langmuir model (Table 3), the theoretical maximum adsorption capacity (*Q_m_*) of NO_3_^−^ were 120.65 mg·g^−1^. The *Q_m_* values and the actual maximum adsorption capacity obtained by the experiments were significantly different. The difference is attributed to the limitations and errors of the model, which are reflected on the isothermal adsorption curve [4]. The *R_L_* values of NO_3_^−^ are between 0 and 1, thus indicating a favorable adsorption process. In the Freundlich model, *K_F_* is related to the adsorption capacity of the adsorbent, which indicates that the adsorption capacity and strength was stable. In the Temkin model, the *R*^2^ values is bigger than 0.8, thus suggesting that the adsorption process includes chemisorption. In the D-R model, *E* represents the chemical adsorption at 8–16 kJ·mol^−1^ and physical adsorption below 8 kJ·mol^−1^ [30]. This indicates that the adsorption of NO_3_^−^ (*E* = 2.52 kJ·mol^−1^) by CB is dominated by physical adsorption. The *Q*_0_ is the maximum unit adsorption capacity, which is associated with the ordinate of last point (Figure 8).

#### 3.3.3. Adsorption Kinetics

**Figure 9:**
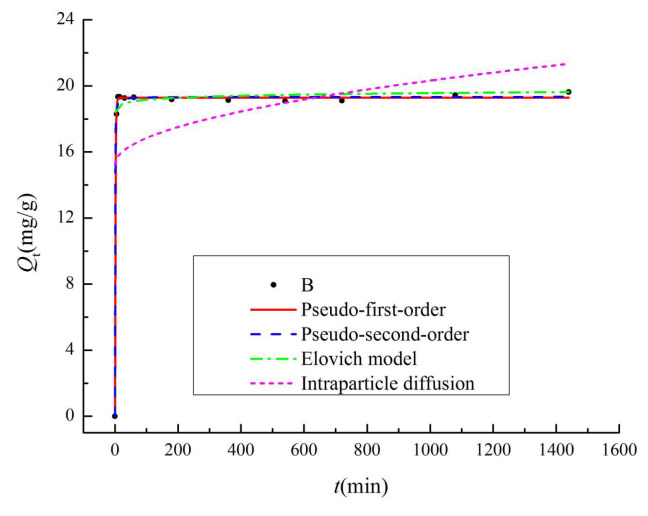
The adsorption kinetics of CB biochar for NO_3_^−^ (650 °C/3 h).

The adsorption kinetics fitting curves are presented in Figure 9, and the kinetic parameters are summarized in Table 4. With increasing adsorption time, the adsorption rate of NO_3_^−^ was fast and large at the beginning, then the curve slowed down and increased slightly, and finally reached adsorption equilibrium. For example, the adsorption capacity reached 92% of the saturated adsorption capacity at 5 min, and gradually tended to equilibrium. The correlation coefficients of the pseudo-second-order model (*R*^2^ > 0.99) were higher than other models, and the fitted equilibrium adsorption capacities were closer to the experimental values. These results suggest that the adsorption of NO_3_^−^ were better described by the pseudo-second-order kinetic model [11]. The adsorption process is a composite adsorption reaction, which includes surface adsorption, external liquid film diffusion, and intraparticle diffusion. This phenomenon can be similarly verified in many reports [26,28,43]. In the pseudo-second-order model (Table 4), *h* represents the initial adsorption rate, and its value is 0.38 mg·g^−1^·min^−1^. The *Q_e_* is very similar in the pseudo-first-order and pseudo-second-order model, both of which are related to the adsorption capacity at an equilibrium concentration. The *R*^2^ values from the Elovich model is larger than 0.9, which indicates that the CB surface adsorption energy is uniformly distributed during the entire adsorption process. For the intraparticle diffusion model, the fitted straight line does not cross the origin, which indicates that particle diffusion is not the only rate-limiting factor [30]. There are other processes (surface adsorption, liquid film diffusion, etc.,) that jointly control the adsorption reaction rate [15]. The *C* are 15.23, which implies the boundary layer of CB has a greater effect on the adsorption processes.

In conclusion, relevant reports on the adsorption of NO_3_^−^ by biochar are summarized in Table 5. Compared to other cellulose- and lignin-based biomass raw materials, the adsorption capacity of NO_3_^−^ by the CB in this study was larger. Even if compared with commercial activated carbon (1.22 mg·g^−1^) [50], the adsorption performance of CB also has certain advantages. Therefore, in view of the preparation temperature and time, the CB can be developed and popularized because of low cost and efficient adsorption performance without complicated modification conditions.

### 3.4. By-Products and Process Analysis

The by-products of *Caragana korshinskii* biomass include bio-oil and tail gas in pyrolysis process. All analytical components of bio-oil and tail gas analyzed by GC-MS are listed in Tables S2 and S3, respectively. After searching in the NIST05 standard mass spectrometry library and consulting relevant data [56], a total of 83 chromatographic peaks were identified in the bio-oil, including 73 compounds in this work. There are 41 chromatographic peaks in the liquid collected by tail gas condensation, including 40 compounds. According to the classification of these organic compounds (Table S2), there are 29 kinds of aliphatic compounds with a relative content of 32.80%, including alkanes, olefins, alcohols, fatty acids, ketones, aldehydes, carbohydrate, etc., 28 aromatic compounds with a relative content of 56.64%, and 16 heterocyclic compounds with a relative content of 10.56%. In general, the content of aromatic compounds in bio-oil is more than the content of aliphatic compounds, and the ratio is 1.73:1. The analysis results in the liquid collected by tail gas condensation (Table S3) are similar to those of bio-oil. The main components of bio-oil analyzed by GC-MS are listed in Table 6 through the screening conditions that the quality matching of GC-MS is greater than 60%. It is worth noting that the content of these compounds accounts for 68.93% of the total, where the higher content is 1,2-cyclopentanedione and 3-methyl-. The content of C6-C15 hydrocarbon compounds in bio-oil is close to 10%, which means that it is of great significance as a potential bio-diesel. The content of ketones and heterocyclic compounds in bio-oils exceeds 20%, which can be used as important chemical raw materials. Benzene and its derivatives and naphthalene are intermediates in the synthesis of spices, plastics, dyes, and drugs [57]. The bio-oil also contains a certain amount of high-value chemicals, such as Guaiacol (Phenol, 2-methoxy-) and α-D-glucose (1,4:3,6-Dianhydro-α-D-glucopyranose). The Guaiacol (relative content 5.08%) is an important fine chemical intermediate and widely used in the synthesis of medicines, spices and dyes [58]. The α-D-glucopyranose (relative content 2.63%), which is mainly used in medical liver function test or medicine, is rarely synthesized artificially considering its cost. Interestingly, the bio-oil contains α-D-glucopyranose and thus can be a valuable product obtained by pyrolysis biomass, which usually exists in the form of black-brown organic liquid mixture. Discussing with some related literature reports [56,57,58], the bio-oil of *Caragana korshinskii* biomass has the advantages of wide raw material sources, abundant reserves, renewable, high energy density, and easy transportation and storage. Khuenkaeo et al. [57] and Lu et al. [58] also believed the bio-oil is a potential source of liquid fuel and chemical raw materials. Therefore, biomass pyrolysis is an important technical method for solving fossil energy problems in the future, and many scholars have reached consensus on this point [56,57,58].

A very interesting process about the change of tail gas is recorded in detail as follows: a slight wood burning odor begins to form at 280 °C, and green smoke is generated at 300 °C, accompanied by an irritating smoke smell. A lot of brown smoke is generated at 350 °C, and the tail gas devices start to collect the bio-oil. The tail gas turns into white smoke at 400 °C followed by a stable smoke flow and a large amount of bio-oil. When the tail gas color becomes lighter at 450 °C, the bio-oil tends to be stable. At 550 °C, the smoke gradually decreases until there is no smoke. No irritating smoke can be smelled at 600 °C. The yield of bio-oil increased with the rise of temperature, and increased greatly from 350 to 450 °C, but slightly from 450 to 550 °C, then gradually decreased to 0 from 550 °C to 600 °C. This result shows that some components in bio-oil further crack to generate small molecules of non-condensable gas, which reduces the yield of bio-oil and further increases the output of non-condensable gas [18]. Up to now, we may not have found any relevant reports on the tail gas color change of pyrolysis biomass.

Therefore, we speculate that the possible mechanism is as follows. The chemical structure of biomass is extremely complex, and its main components are cellulose, hemicellulose, and lignin. Among them, the pyrolysis mechanism of cellulose has been widely studied and a preliminary theoretical system has been formed [59]. However, hemicellulose and lignin are relatively lacking on their pyrolysis mechanism because of their complex structure and many research branches. In general, during the pyrolysis process, water is evaporated at 105 °C, and the main pyrolysis temperature of cellulose is 240–375 °C, which usually produces dehydrated fiber and L-glucose. Hemicellulose softens and decomposes at 200–295 °C and usually produces volatile products. Lignin decomposes at 280–500 °C, usually producing carbon and various organic compounds. From the perspective of matter and energy, heat is first transferred to the biomass surface and then the inside. The pyrolysis process proceeds layer by layer from the inside to the outside, and the heated part of the biomass is rapidly decomposed into biochar and volatiles [60]. Among them, the volatile matter is composed of condensable gas and non-condensable gas, and the condensable gas is rapidly condensed to obtain bio-oil [57]. With the transfer of heat, the organic matter inside the biomass is heated to continue pyrolysis, and part of the primary pyrolysis product undergoes a second pyrolysis or multiple pyrolysis until it is stable.

## 4. Conclusions

This work used *Caragana korshinskii* biomass as a raw material to prepare 15 kinds of biochar by controlling the oxygen-limited pyrolysis process. On the whole, the effect of pyrolysis temperature on the CB properties is the most important, which is greater than pyrolysis time. The CEC, pH, and BET specific surface area of CB under each pyrolysis process were 16.64–81.4 cmol·kg^−1^, 6.65–8.99, and 13.52–133.49 m^2^·g^−1^, respectively. With the increase of pyrolysis temperature and time, the bond valence structure of C 1s, Ca 2p, and O 1s is more stable, and the phase structure of CaCO_3_ is more obvious. The surface of CB biochar contains abundant functional groups, such as hydroxyl, carboxyl, ester carbonyl, NH_4_^+^, pyridine, etc. With the increase of pyrolysis temperature, the content of basic functional groups (benzene ring and pyridine) and the aromaticity increases, while the content of acidic functional groups (phenolic hydroxyl and carboxyl) decreases and the polarity decrease. The CB prepared at 650 °C for 3 h presented the best adsorption performance, and the maximum theoretical adsorption capacity for NO_3_^−^ reached 120.65 mg·g^−1^. The Langmuir model and pseudo-second-order model can well describe the isothermal and kinetics process of NO_3_^−^, respectively. This indicated that the beneficial adsorption process via monolayer was affected by surface adsorption, intraparticle diffusion and liquid film diffusion, and the physical adsorption is the more important mechanism. The by-products contain bio-soil and tail gas, which are potential source of liquid fuel and chemical raw materials. The bio-oil of CB contains α-d-glucopyranose, which can be used in medical tests and medicines. The color and flow rate of the tail gas also change correspondingly with the increase of temperature during the pyrolysis process. Therefore, the CB and bio-oil from *Caragana korshinskii* biomass will serve arid areas of Asia and Africa as innovative products, and CB is worth to be further developed and popularized because of its low cost and efficient adsorption performance without complicated modification condition.

## Figures and Tables

**Table 1:** The list of kinetic and isotherm models.

Models	Expression	Parameters
Langmuir	$Q_{e}=\frac{aQ_{m}C_{e}}{1+aC_{e}}$, $R_{L}=\frac{1}{1+aC_{0}}$	*Q_m_*, *a*
Freundlich	*Q_e_* = *K_F_C_e_^1/n^*	*K_F_*, 1/*n*
Temkin	*Q_e_* = *B*ln*A* + *B*ln*C_e_*	*A*, *B*
Dubinin-Radushkevich (D-R) model	$\ln Q_{e}=\ln Q_{0}-\beta \varepsilon^{2}$, $\varepsilon =RT\ln(1+\frac{1}{C_{e}})$ $E=\frac{1}{{\left(2\beta \right)}^{0.5}}$	*Q*_0_, *β*, *ε,E*
Pseudo-first-order	$Q_{t}=Q_{e}(1-\exp(-\frac{k_{1}}{2.303}t))$	*Q_e_*, *k*_1_
Pseudo-second-order	$Q_{t}=\frac{k_{2}Q_{e}{}^{2}t^{}}{1+k_{2}Q_{e}t}$, $h=k_{{}^{2}}Q_{e}{}^{2}$	*Q_e_*, *k*_2_, *h*
Elovich model	$Q_{t}=\frac{1}{b}\ln(a^{\prime }bt+1)$	*a*′, *b*
Intraparticle diffusion	$Q_{t}=k_{i}t^{0.5}+C$	*C*, *k_i_*

**Table 2:** Basic physical and chemical properties of biochar produced from *Caragana korshinskii*.

biochar	C (%)	O (%)	N (%)	O/C	N/C	(O + N)/C	pH	CEC (cmol·kg^−1^)	Yield (%)
CB450(2h)	77.99	16.18	3.10	0.21	0.04	0.25	6.65	62.51	33.12
CB500(2h)	78.25	15.6	2.05	0.2	0.03	0.23	8.55	66.12	33.00
CB550(2h)	77.9	13.52	1.26	0.17	0.02	0.19	8.80	35.00	28.93
CB600(2h)	82.17	12.52	1.10	0.15	0.01	0.17	8.96	16.64	25.94
CB650(2h)	85.1	10.04	0.84	0.12	0.01	0.13	8.93	46.44	27.24
CB450(3h)	65.73	22.9	2.79	0.35	0.04	0.39	8.04	55.72	33.23
CB500(3h)	74.55	18.73	2.41	0.25	0.03	0.28	7.72	74.42	31.80
CB550(3h)	77.02	16.84	2.13	0.22	0.03	0.25	8.84	31.97	31.43
CB600(3h)	79.12	13.19	1.47	0.17	0.02	0.19	8.97	20.48	28.20
CB650(3h)	85.31	10.59	0.72	0.12	0.01	0.13	8.99	38.93	23.08
CB450(4h)	74.35	18.17	3.28	0.24	0.04	0.29	7.51	81.40	30.31
CB500(4h)	72.13	18.83	2.95	0.26	0.04	0.30	8.74	44.62	29.78
CB550(4h)	81.42	11.52	2.14	0.14	0.03	0.17	8.99	32.91	28.65
CB600(4h)	79.79	12.92	1.5	0.16	0.02	0.18	8.68	30.66	27.56
CB650(4h)	81.04	10.86	0.62	0.13	0.01	0.14	8.97	17.75	27.38

**Table 3:** Isothermal adsorption fitting parameters of CB for NO_3_^−^.

Models	Langmuir				Freundlich			Temkin			D-R Model		
	*a*	*Q_m_* (mg·g^−1^)	*R_L_*	*R* ^2^	*K_F_*	*n*	*R* ^2^	*A*	*B*	*R* ^2^	*Q*_0_ (mmol·g^−1^)	*E* (kJ·mol^−1^)	*R* ^2^
CB (650 °C/3 h)	0.02	120.65	0.0008-0.97	0.9364	2.45	1.20	0.9355	0.77	10.21	0.8506	33.04	2.52	0.8568

**Table 4:** Adsorption kinetics fitting parameters of CB for NO_3_^−^.

Models	Pseudo-First-Order			Pseudo-Second-Order				Elovich			Intraparticle Diffusion		
	*Q_e_* (mg·g^−1^)	*k*_1_	*R* ^2^	*Q_e_* (mg·g^−1^)	*k*_2_	*H* (mg·g^−1^·min^−1^)	*R* ^2^	*a*′	*b*	*R* ^2^	*k_i_*	*C*	*R* ^2^
CB(650 °C/3 h)	19.28	1.37	0.9991	19.45	0.001	0.38	0.9998	4.68	5.70	0.9959	0.16	15.23	0.06

**Table 5:** Comparison of sorption capacity of CB with selected biochars derived from other materials for NO_3_^−^.

Raw Materials	Temperature (°C) ^1^	Time (h) ^1^	Maximum Adsorption Capacity (mg·g^−1^) ^2^	Literature
Wheat straw	300	1	1.10	[50]
Mustard straw	300	1	1.30	[50]
Ponderosa pine wood	650	0.3	6.20	[51]
Switchgrass	650	0.3	1.84	[51]
Corn stover	650	0.3	6.25	[51]
Sugar beet bagasse	700	1.5	9.14	[52]
Rice husk	600	-	2.1	[53]
Date palm	700	4	8.37	[54]
Fe-impregnated corn stalk	550	0.5	15.41	[29]
ZnCl_2_ activated Sugar beet bagasse	700	1.5	27.55	[52]
Amine-grafted corn cob	100	24	49.9	[55]
Amine-grafted coconut copra	100	48	59.2	[55]
*Caragana korshinskii*	650	3	120.65	This work

^1^ Pyrolysis temperature and time. ^2^ the maximum adsorption capacity in the table is the maximum of the actual adsorption capacity or the theoretical maximum adsorption capacity calculated by Langmuir model.

**Table 6:** The main components of bio-oil analyzed by GC-MS.

Number	Library (ID)	CAS	Quality (%)	Peak Area (%)
1	2-Furanmethanol	000098-00-0	90	2.40
2	Pyridine, 3-methyl-	000108-99-6	92	1.22
3	2,6-Lutidine	000108-48-5	70	0.39
4	2-Cyclopenten-1-one, 2-methyl-	001120-73-6	80	1.73
5	Pyridine, 3,5-dimethyl-	000591-22-0	90	0.80
6	Pyridine, 2,3-dimethyl-	000583-61-9	94	0.50
7	2-Cyclopenten-1-one, 3-methyl-	002758-18-1	91	2.49
8	Phenol	000108-95-2	91	4.24
9	3-Methylpyridazine	001632-76-4	64	0.22
10	Pyridine, 3-methoxy-	007295-76-3	80	0.54
11	1,2-Cyclopentanedione, 3-methyl-	000765-70-8	95	6.70
12	2-Cyclopenten-1-one, 2,3-dimethyl-	001121-05-7	90	1.18
13	Phenol, 2-methyl-	000095-48-7	97	2.12
14	p-Cresol	000106-44-5	95	5.51
15	Phenol, 2-methoxy-	000090-05-1	94	5.08
16	Phenol, 2,6-dimethyl-	000576-26-1	76	0.12
17	Maltol	000118-71-8	62	0.17
18	2-Cyclopenten-1-one, 3-ethyl-2-hydroxy-	021835-01-8	97	1.67
19	Phenol, 2,4-dimethyl-	000105-67-9	95	2.92
20	Phenol, 4-ethyl-	000123-07-9	93	0.78
21	Naphthalene	000091-20-3	87	1.39
22	Creosol	000093-51-6	97	3.37
23	1,4:3,6-Dianhydro-.alpha.-d-glucopyranose	1000098-14-8	95	2.63
24	Phenol, 4-ethyl-3-methyl-	001123-94-0	64	0.75
25	1,2-Benzenediol, 3-methoxy-	000934-00-9	94	2.33
26	4-Methoxybenzene-1,2-diol	003934-97-2	62	1.27
27	Phenol, 4-ethyl-2-methoxy-	002785-89-9	90	2.70
28	Naphthalene, 2-methyl-	000091-57-6	97	2.33
29	Phenol, 2,6-dimethoxy-	000091-10-1	96	6.54
30	Naphthalene, 1,3-dimethyl-	000575-41-7	94	0.69
31	Naphthalene, 1,5-dimethyl-	000571-61-9	95	0.61
32	3,5-Dimethoxy-4-hydroxytoluene	006638-05-7	96	3.51

## Supplementary Materials

The following are available online at https://www.mdpi.com/1996-1944/13/15/3391/s1, Table S1: The surface characteristics of alternative CB biochar and Caragana korshinskii, Table S2: The all components of bio-oil analyzed by GC-MS, Table S3: The all components of the liquid collected by tail gas condensation analyzed, Figure S1: Scanning electron microscope (SEM) images of Energy Dispersive Spectrometer (EDS) spectra of CB at 650 °C/3 h, Figure S2: BJH (Barren-Joyner-Halenda)-adsorption-pore size distribution of CB at 650 °C/3 h.
