# Supplementary material for: The Eco-Friendly Biochar and Valuable Bio-Oil from Caragana korshinskii: Pyrolysis Preparation, Characterization, and Adsorption Applications
Source: Materials (Basel). 2020 Jul 31;13(15):3391. doi: 10.3390/ma13153391 (PMC7435931; doi:10.3390/ma13153391)
Supplement: Supplementary file 1 [file materials-13-03391-s001.pdf]

materials

Supplementary Materials: The Eco-Friendly Biochar and Valuable Bio-Oil from *Caragana korshinskii*: Pyrolysis Preparation, Characterization, and Adsorption Applications

Tongtong Wang [1,2], Hongtao Liu [1,2], Cuihua Duan [1,2], Rui Xu [1,2], Zhiqin Zhang [1,2], Diao She [1,3] and Jiyong Zheng [1,3,*]

[1] State Key Laboratory of Soil Erosion and Dryland Farming on the Loess Plateau, Northwest A & F University, Yangling, Shaanxi 712100, China; tongtwang@163.com (T.W.); liuhongtao@nwafu.edu.cn (H.L.); chduan@nwafu.edu.cn (C.D.); 2019055450@nwafu.edu.cn (R.X.); zhangzhiqin@nwafu.edu.cn (Z.Z.); diaoshe888@163.com (D.S.)
[2] College of Natural Resources and Environment, Northwest A & F University, Yangling, Shaanxi 712100, China
[3] Institute of Soil and Water Conservation, Chinese Academy of Sciences and Ministry of Water Resources, Yangling, Shaanxi 712100, China
* Correspondence: zhjy@ms.iswc.ac.cn; Tel.: +86-1502-928-2338

Table S1. The surface characteristics of alternative CB biochar and *Caragana korshinskii*.

Surface Feature	Test Item	Unit	Type: CB (650 °C, 3h)	Type: *Caragana korshinskii*	Remarks
Surface Area	Single point surface area	$m^2 \cdot g^{-1}$	127.015	2.870	Relative pressure (P/P_0) = 0.250; Where P is the adsorption pressure and P_0 is the saturated vapor pressure of the adsorbate
	BET Surface Area		133.491	1.917	Data fetching range 0.055~0.201
	Langmuir Surface Area		175.947	2.851	Monolayer adsorption model calculation
	BJH Adsorption cumulative surface area		14.655	21.053	The aperture range is 2~55
	BJH Desorption cumulative surface area		3.211	13.915	The aperture range is 2~55
Pore Volume	Single point adsorption total pore volume	$cm^3 \cdot g^{-1}$	0.139	0.025	When P/P_0 = 0.964, the total pore volume smaller than the critical pore diameter 55
	BJH Adsorption cumulative volume		0.088	0.034	The aperture range is 2~55
	BJH desorption cumulative volume		0.075	0.024	The aperture range is 2~55
Pore Size	Total adsorption average pore width	nm	4.169	-	Calculated from $4V/A$, where the A corresponds to the adsorption BET specific surface area, adsorption cumulative pore internal surface area, desorption cumulative pore internal surface
	BJH Adsorption average pore width		24.036	-	

BJH Desorption average pore width	109.712	-	area, respectively; *V* is the gas adsorption volume

Table S2. The all components of bio-oil analyzed by GC-MS.

Peak Number	Library/ID	CAS	Quality (%)	Peak Area (%)
1	Pentanoic acid, 3-methyl-	000105-43-1	33	0.12
2	2-Furanmethanol	000098-00-0	90	2.40
3	Pyridine, 3-methyl-	000108-99-6	92	1.22
4	2,6-Lutidine	000108-48-5	70	0.39
5	1,3-Cyclopentadiene, 5-(1-methylethylidene)-	002175-91-9	25	0.52
6	2-Cyclopenten-1-one, 2-methyl-	001120-73-6	80	1.73
7	R(-)-1-Cyano-2-methylpyrrolidine	1000145-01-8	35	2.65
8	Pyridine, 3,5-dimethyl-	000591-22-0	90	0.80
9	Pyridine, 2,3-dimethyl-	000583-61-9	94	0.50
10	2-Cyclopenten-1-one, 3-methyl-	002758-18-1	91	1.30
11	2-Cyclopenten-1-one, 3-methyl-	002758-18-1	72	1.19
12	Pyridine, 1-oxide	000694-59-7	35	0.13
13	Phenol	000108-95-2	91	4.09
14	Phenol	000108-95-2	80	0.14
15	3-Methylpyridazine	001632-76-4	64	0.22
16	Cyclohexene, 1,6-dimethyl-	001759-64-4	42	0.84
17	Pyrazine, 2-ethyl-5-methyl-	013360-64-0	47	0.79
18	Pyridine, 3-methoxy-	007295-76-3	80	0.54
19	Succinic acid, 3-methylbut-2-en-1-yl 2,3-dimethylphenyl ester	1000390-01-8	35	1.67
20	Cyclotetrasiloxane, octamethyl-	000556-67-2	43	1.00
21	Cyclopentene, 1-(1-methylethyl)-	001462-07-3	49	0.20
22	1,2-Cyclopentanedione, 3-methyl-	000765-70-8	95	6.70
23	2-Cyclopenten-1-one, 2-hydroxy-3-methyl-	000080-71-7	55	0.31
24	2-Cyclopenten-1-one, 2,3-dimethyl-	001121-05-7	90	1.18
25	2-Cyclopenten-1-one, 2-hydroxy-3,4-dimethyl-	021835-00-7	58	0.32
26	Phenol, 2-methyl-	000095-48-7	97	2.12
27	Octane, 3-ethyl-2,7-dimethyl-	062183-55-5	59	1.71
28	p-Cresol	000106-44-5	95	5.29
29	p-Cresol	000106-44-5	55	0.22
30	Phenol, 2-methoxy-	000090-05-1	94	5.08
31	2,5-Pyrrolidinedione, 1-methyl-	001121-07-9	53	0.94
32	1,2-Benzenedimethanol	000612-14-6	22	0.22
33	Phenol, 2,6-dimethyl-	000576-26-1	76	0.12
34	2H-Azepin-2-one, hexahydro-1-methyl-	002556-73-2	59	0.34
35	Maltol	000118-71-8	62	0.17
36	2-Cyclopenten-1-one, 3-ethyl-2-hydroxy-	021835-01-8	97	1.67
37	5,5-Dimethyl-3-oxo-1-pyrroline, 1-oxide	1000305-98-3	47	0.31
38	Phenol, 3-ethyl-	000620-17-7	50	0.96
39	Benzene, 4-ethyl-1,2-dimethyl-	000934-80-5	45	0.41
40	Phenol, 2,4-dimethyl-	000105-67-9	95	2.92
41	1H-Imidazole, 4-methyl-5-nitro-	014003-66-8	25	0.11
42	Phenol, 4-ethyl-	000123-07-9	93	0.78
43	Naphthalene	000091-20-3	87	1.39
44	Creosol	000093-51-6	97	3.37
45	1-Bromo-8-tetrahydropyranyloxyoctane	050816-20-1	32	0.43
46	Phenol, 4-ethyl-	000123-07-9	46	0.40
47	1,4:3,6-Dianhydro-.alpha.-d-glucopyranose	1000098-14-8	95	2.63
48	Propionic acid, 2,2-dichloro-, pentyl ester	017640-08-3	47	0.69
49	2,3-Anhydro-d-mannosan	1000129-98-0	45	2.01
50	Catechol	000120-80-9	49	0.17

51	Catechol	000120-80-9	53	0.32
52	Catechol	000120-80-9	52	0.60
53	Cyclohexene, 3-methyl-6-(1-methylethylidene)-	000586-63-0	41	0.34
54	Catechol	000120-80-9	43	0.24
55	2-Propanone, 1-(N-cyanomethylimino-)	1000222-11-8	38	0.17
56	Phenol, 4-ethyl-3-methyl-	001123-94-0	64	0.75
57	1-(1-Bromo-2-(phenylthio)cycloprop-1-yl)cyclopentan-1-ol	1000139-16-7	27	0.29
58	1,2-Benzenediol, 3-methoxy-	000934-00-9	94	1.09
59	1,2-Benzenediol, 3-methoxy-	000934-00-9	93	0.30
60	1,2-Benzenediol, 3-methoxy-	000934-00-9	94	0.77
61	1,2-Benzenediol, 3-methoxy-	000934-00-9	76	0.17
62	4-Methoxybenzene-1,2-diol	003934-97-2	62	1.27
63	o-Methoxybenzonitrile	006609-56-9	18	1.22
64	Phenol, 4-ethyl-2-methoxy-	002785-89-9	90	2.70
65	Naphthalene, 2-methyl-	000091-57-6	97	1.61
66	Naphthalene, 2-methyl-	000091-57-6	93	0.72
67	5,6,7,8-Tetrahydroquinoxaline	034413-35-9	30	0.65
68	Phenol, 2,6-dimethoxy-	000091-10-1	96	6.54
69	Benzenemethanol, 3-hydroxy-	000620-24-6	46	0.98
70	2,4-Dimethoxy-5-methyl pyrimidine	005151-34-8	35	0.86
71	Phenol, 2-methoxy-4-propyl-	002785-87-7	49	0.62
72	Naphthalene, 2,6-dimethyl-	000581-42-0	52	0.65
73	2-Decanol, trifluoroacetate	1000352-32-8	35	0.41
74	Naphthalene, 1,3-dimethyl-	000575-41-7	94	0.69
75	Naphthalene, 1,5-dimethyl-	000571-61-9	95	0.61
76	3,5-Dimethoxy-4-hydroxytoluene	006638-05-7	96	3.51
77	Undecane	001120-21-4	27	0.02
78	5-tert-Butylpyrogallol	020481-17-8	53	3.41
79	2-Mercaptobenzothiazole	000149-30-4	38	0.61
80	Furaldehyde phenylhydrazone	002216-75-3	53	0.59
81	Phenol, 2,6-dimethoxy-4-(2-propenyl)-	006627-88-9	58	0.72
82	1-(1-Hydroxybutyl)-2,5-dimethoxybenzene	149083-03-4	38	0.64
83	2-Decenal, (E)-	003913-81-3	53	0.47

Table S3. The all components of the liquid collected by tail gas condensation analyzed.

Peak Number	Library/ID	CAS	Quality (%)	Peak Area (%)
1	Butanoic acid, 3-oxo-, 1-methylpropyl ester	013562-76-0	59	0.95
2	2-Furanmethanol	000098-00-0	83	3.52
3	Pyridine, 3-methyl-	000108-99-6	76	5.69
4	2,6-Lutidine	000108-48-5	68	1.22
5	2-Cyclopenten-1-one, 2-methyl-	001120-73-6	74	2.43
6	Pyrazine, 2,6-dimethyl-	000108-50-9	30	4.72
7	Pyridine, 2,5-dimethyl-	000589-93-5	76	1.75
8	2-Cyclopenten-1-one, 3-methyl-	002758-18-1	86	1.01
9	2-Cyclopenten-1-one, 3-methyl-	002758-18-1	74	1.85
10	Phenol	000108-95-2	91	3.21
11	3-Methylpyridazine	001632-76-4	42	0.37
12	N-.beta.-Hydroxyethylsalicylaldehyde hydrazone	086547-03-7	38	1.26
13	Pyridine, 3-methoxy-	007295-76-3	38	3.14
14	3-Pyridinecarboxylic acid, 1,2-dihydro-4,6-dimethyl-2-oxo-, ethyl ester	1000349-78-0	35	1.12
15	1,2-Cyclopentanedione, 3-methyl-	000765-70-8	94	8.34
16	2-Cyclopenten-1-one, 2,3-dimethyl-	001121-05-7	72	1.27
17	3-Heptene, (E)-	014686-14-7	30	0.39
18	Phenol, 3-methyl-	000108-39-4	90	1.60

19	Phytol	000150-86-7	23	1.55
20	Phenol, 3-methyl-	000108-39-4	95	4.65
21	p-Cresol	000106-44-5	30	0.41
22	Ethanone, 1-(2-methyl-1-cyclopenten-1-yl)-	003168-90-9	90	5.73
23	2,5-Pyrrolidinedione, 1-methyl-	001121-07-9	52	6.05
24	Carbonic acid, decyl prop-1-en-2-yl ester	1000382-90-5	10	0.40
25	Cyclopentane, 1-acetyl-1,2-epoxy-	015121-02-5	38	2.14
26	1H-Imidazole-4-carboxylic acid, methyl ester	017325-26-7	72	0.35
27	2-Dodecyne	000629-49-2	32	1.59
28	1-(2-Diethylaminoethyl)-2,3,4,5,6,7-hexahydro-4-oxo-1H-cyclopenta[b]pyridine	018121-17-0	27	2.42
29	Decanal	000112-31-2	14	1.92
30	Creosol	000093-51-6	55	5.51
31	1,4:3,6-Dianhydro- alpha.-d-glucopyranose	1000098-14-8	91	8.22
32	2-Piperidinone, N-(4-bromo-n-butyl)-	195194-80-0	47	0.94
33	6-Methyl-1,5-diazabicyclo[3.1.0]hexane	100463-00-1	35	1.81
34	4-Fluoro-3-methylanizole	1000342-42-1	46	0.90
35	2-Methoxy-4-methyl-bicyclo[3.2.1]oct-2-ene	1000188-09-4	49	1.48
36	Hydroquinone	000123-31-9	46	1.25
37	2(1H)-Pyrimidinone, 5-methyl-	041398-85-0	47	0.66
38	1-Aza-2-boracyclopentane, 2-ethyl-1-methyl-	1000149-42-8	59	0.97
39	Phenol, 2,6-dimethoxy-	000091-10-1	95	5.71
40	3,5-Dimethoxy-4-hydroxytoluene	006638-05-7	68	1.63
41	3-Isopropyl-1-methyl-4-methylamino-pyrrole-2,5-dione	1000296-12-2	38	0.12

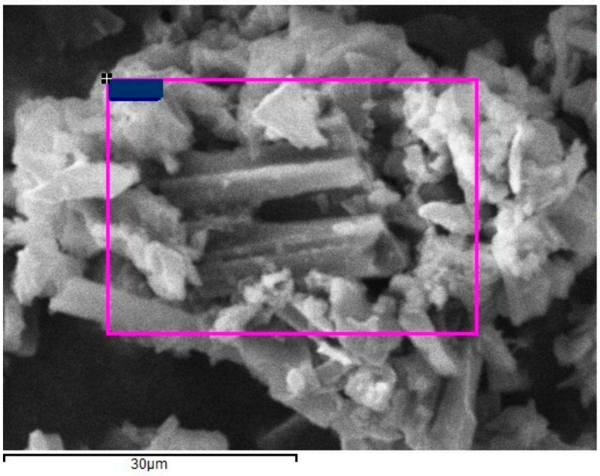

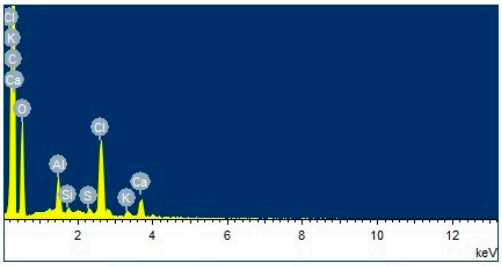

element	content %	atom %
C K	74.59	81.82
O K	19.19	15.8
Si K	0.14	0.07
Cl K	3.49	1.3
K K	0.3	0.1
Ca K	1.19	0.39

Figure S1. Scanning electron microscope (SEM) images of Energy Dispersive Spectrometer (EDS) spectra of CB at 650 °C/3h.

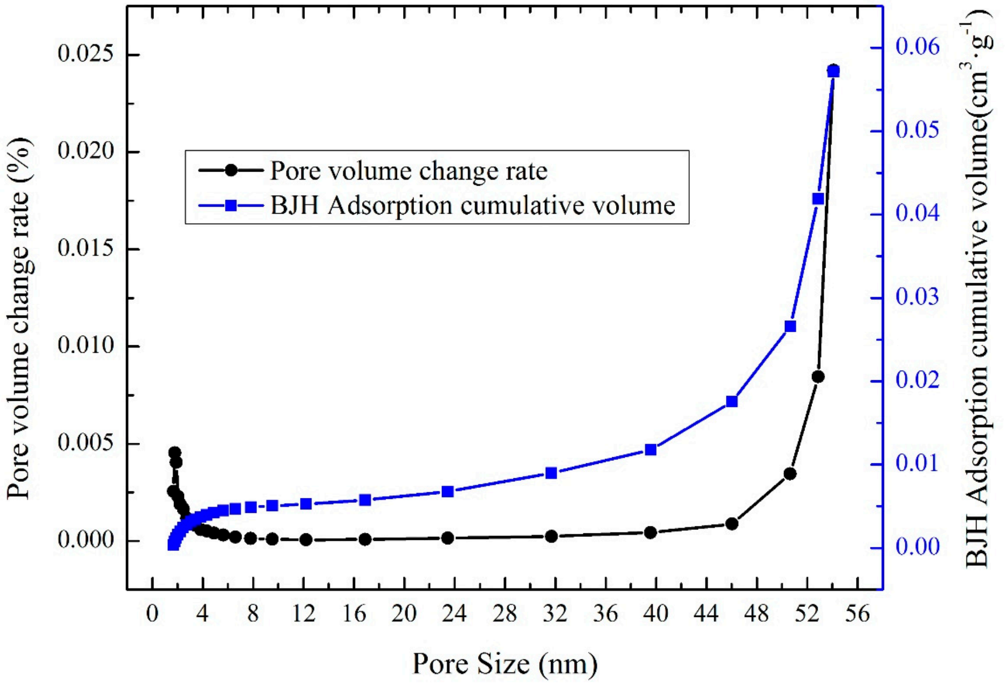

Figure S2. BJH (Barren-Joyner-Halenda)-adsorption-pore size distribution of CB at 650 °C/3h.

© 2020 by the authors. Submitted for possible open access publication under the terms and conditions of the Creative Commons Attribution (CC BY) license (http://creativecommons.org/licenses/by/4.0/).
